# Supplementary material for: Clinical judgment model-based nursing simulation scenario for patients with upper gastrointestinal bleeding: A mixed methods study
Source: PLoS One. 2021 May 3;16(5):e0251029. doi: 10.1371/journal.pone.0251029 (PMC8092762; doi:10.1371/journal.pone.0251029)
Supplement: S1 File — (DOCX) [file pone.0251029.s001.docx]

**연구 대상자 사전 일반적 특성관련 설문지**

| - 자기결정성 학습동기 -  이 검사는 학교와 가정에서 공부에 임하는 학습동기를 알아보기 위한 것입니다. 각 문항은 **옳고 그른 답이 없으므로 문제를 잘 읽고 자신과 가장 가깝다고 생각하는** 빈칸에 O표나 √표를 하세요 | | | | | | |
| --- | --- | --- | --- | --- | --- | --- |
| 문항 | 내용 | 전혀  그렇지않다 | 대체로 그러지 않다 | 보통  이다 | 대체로  그렇다 | 매우  그렇다 |
|  |  | 1 | 2 | 3 | 4 | 5 |
| 1 | 학교에서 배우는 과목들은 내가 공부해야만 하는 중요한 것이기 때문에 |  |  |  |  |  |
| 2 | 문제를 푸는 것이 흥미롭기 때문에 |  |  |  |  |  |
| 3 | 간호사 국가고시와 관련된 중요한 교과들이기 때문에 |  |  |  |  |  |
| 4 | 어려운 도전들로부터 기쁨을 얻기 때문에 |  |  |  |  |  |
| 5 | 나는 공부하는 것을 삶의 한 부분으로서 필요하다고 생각하기 때문에 |  |  |  |  |  |
| 6 | 공부를 잘 하게 되는 것이 즐거우니까 |  |  |  |  |  |
| 7 | 새로운 것을 알아가길 원하기 때문에 |  |  |  |  |  |
| 8 | 나는 생각하는 것을 좋아하기 때문에 |  |  |  |  |  |
| 9 | 공부하는 것이 미래의 나를 위해 유용하기 때문에 |  |  |  |  |  |
| 10 | 내가 나의 지식을 키우는 것이 재미있어서 |  |  |  |  |  |
| 11 | 내가 이해하고 싶은 교과이기 때문에 |  |  |  |  |  |
| 12 | 실패와 실수를 극복하는 것에 보람을 느끼기 때문에 |  |  |  |  |  |

| - 메타인지 척도 -  이 검사는 평소 여러분이 학습을 하거나 과제를 수행하는 동안 무엇을 생각하며 어떻게 접근하는지 또 수행과정을 어떻게 통계하는지와 관련하여 자주 활용하는 학습방법을 알아보기 위한 것(=메타인지 척도) 입니다.각 문항은 **옳고 그른 답이 없으므로** 문제를 잘 읽고 **자신의 생각과 가장 가깝다고 생각하는** 빈칸에 O표나 √표를 하세요. | | | | | | |
| --- | --- | --- | --- | --- | --- | --- |
| 문항 | 내용 | 전혀  그렇지않다 | 대체로 그러지 않다 | 보통  이다 | 대체로  그렇다 | 매우  그렇다 |
|  |  | 1 | 2 | 3 | 4 | 5 |
| 1 | 나는 공부를 시작하기 전에 무엇을 어떻게 공부할 지 미리 머릿속으로 생각해 본다. |  |  |  |  |  |
| 2 | 나는 공부에 집중하다가도 현재 내가 배워야 할 내용이 무엇인지 스스로에게 물어볼 때가 있다. |  |  |  |  |  |
| 3 | 나는 책을 읽을 때 시간이 부족하면 중요한 부분만 찾아 읽는다. |  |  |  |  |  |
| 4 | 나는 무엇부터 공부할지 순서를 정한 후 시작한다. |  |  |  |  |  |
| 5 | 나는 공부하는 도중에 내용을 확실히 이해하고 있는지 수시로 점검해 본다. |  |  |  |  |  |
| 6 | 나는 학습 내용과 양에 따라 시간을 분배하여 공부한다. |  |  |  |  |  |
| 7 | 나는 공부를 시작하기 전에 공부할 양을 미리 정한다. |  |  |  |  |  |
| 8 | 나는 문제집을 풀어 본 후 스스로 채점하고 틀린 문제는 다시 공부한다. |  |  |  |  |  |
| 9 | 나는 책을 읽을 때 시간이 부족하면 중요하지 않은 부분은 건너뛴다. |  |  |  |  |  |
| 10 | 나는 시험을 보기 전 미리 계획을 세워서 공부한다. |  |  |  |  |  |
| 11 | 나는 숙제를 한 뒤 잘 했는지 다시 한번 확인한다. |  |  |  |  |  |
| 12 | 내가 잘 못하거나 자신없는 과목은 시간을 늘려 공부한다. |  |  |  |  |  |
| 13 | 나는 공부하는 시간과 노는 시간을 정해놓고 실천한다. |  |  |  |  |  |
| 14 | 나는 시험을 볼 때 다 풀고 나서 다시 한번 확인해 본다. |  |  |  |  |  |
| 15 | 나는 공부할 때 이해가 잘 되지 않는 내용은 다시 공부한다. |  |  |  |  |  |
| 16 | 나는 시험공부를 하다가 시간이 모자라면 중요한 부분만 찾아서 공부한다. |  |  |  |  |  |
| 17 | 학년말 시험 공부를 할 때 그전에 보았던 시험지를 다시 찾아 공부한다. |  |  |  |  |  |
| 18 | 나는 문제가 잘 풀리지 않으면 다른 방법으로 풀어본다. |  |  |  |  |  |
| 19 | 나는 시간 계획을 세우지 않고 공부하면 학습 능률이 오르지 않는다. |  |  |  |  |  |
| 20 | 나는 문제를 풀 때 잘 모르면 교과서를 다시 찾아본다. |  |  |  |  |  |
| 21 | 이해가 잘 되지 않는 내용은 여러 번 읽고 생각해본다. |  |  |  |  |  |
| 22 | 나는 시험 공부를 할 때 과목별로 시간 계획을 세워 공부한다. |  |  |  |  |  |
| 23 | 나는 시험을 본 후 틀린 문제를 집중적으로 공부한다. |  |  |  |  |  |
| 24 | 나는 시험 공부를 할 때 어려운 과목은 더 주의를 기울여 공부한다. |  |  |  |  |  |
| 25 | 나는 어디까지 얼마동안 공부할 것인지 계획을 세워 공부를 시작한다. |  |  |  |  |  |
| 26 | 나는 수업 시간에 배우고 있는 내용을 이해하고 있는지 다시 한 번 머리 속으로 떠올려 본다. |  |  |  |  |  |
| 27 | 나는 공부를 하다가 잘 모르는 부분이 생기면 앞부분의 관련된 내용을 다시 천천히 읽어본다. |  |  |  |  |  |
| 28 | 나는 효과적으로 공부하기 위해 시간마다 계획을 세워서 공부한다. |  |  |  |  |  |
| 29 | 내가 계획한 대로 시험공부를 하고 있는지 스스로 반성해 본다. |  |  |  |  |  |
| 30 | 나는 수업 시간에 이해가 잘 되지 않는 부분을 표시해 두었다가 나중에 공부한다. |  |  |  |  |  |

| - 비판적 사고성향 -  이 검사는 평소 여러분의 비판적 사고성향을 측정하기 위한 것입니다. 각 문항은 **옳고 그른 답이 없으므로** 문제를 잘 읽고 **자신과 가장 가깝다고 생각하는** 빈칸에 O표나 √표를 하세요 | | | | | |
| --- | --- | --- | --- | --- | --- |
| 문항 | 전혀  그렇지  않다 | 그렇지  않다 | 그저  그렇다 | 그렇다 | 매우  그렇다 |
| 1. 나는 어떤 결정을 할 때 충분히 생각하지 않고 속단하는 경향이 있다. |  |  |  |  |  |
| 2. 나는 내 신념에 대해 나름대로 근거를 가지고 있다. |  |  |  |  |  |
| 3. 나는 만약 내가 확실하게 잘못한 것이 있다면 기꺼이 인정한다. |  |  |  |  |  |
| 4. 나는 어떤 상황이 내 생각과 다르더라도 사실이라고 밝혀지면 그것을 받아드린다. |  |  |  |  |  |
| 5. 나는 타당하고 충분한 근거가 확보될 때까지 판단을 보류하고 심사숙고 한다. |  |  |  |  |  |
| 6. 나는 어떤 주장이든 그것을 뒷받침할 수 있는 타당한 근거가 필요하다고 생각 한다. |  |  |  |  |  |
| 7. 나는 일상적으로 하던 일도 새로운 것처럼 다시 생각해 볼 때가 많다. |  |  |  |  |  |
| 8. 나는 다른 사람의 의견에 동의하지 않을 때 그 이유를 설명 한다. |  |  |  |  |  |
| 9. 나는 어떤 결론을 내릴 때 시작부터 끝까지 대체로 논리적이다. |  |  |  |  |  |
| 10. 나는 책에 있는 내용이라도 의문이 생길 때가 있다. |  |  |  |  |  |
| 11. 다른 사람이 나를 평할 때 논리적이라고 한다. |  |  |  |  |  |
| 12. 내가 옳다고 믿는 것들이 혹시 잘못된 것은 아닐까 하는 생각을 할 때가 있다. |  |  |  |  |  |
| 13. 나는 모르는 것을 알기 위해 열성적으로 노력 한다. |  |  |  |  |  |
| 14. 나는 풀어야 할 문제가 있을 때 체계적으로 문제 해결과정을 적용 한다. |  |  |  |  |  |
| 15. 나는 어려운 일도 스스로 헤쳐 나갈 수 있다고 생각한다, |  |  |  |  |  |
| 16. 나는 사람들이 당연하다고 인정하는 것에도 종종 의문이 생긴다. |  |  |  |  |  |
| 17. 나는 복잡한 문제라도 기꺼이 풀어 나가려고 애쓴다. |  |  |  |  |  |
| 18. 나는 빨리 판단하지 않고 거듭 생각하는 편이다. |  |  |  |  |  |
| 19. 나는 복잡하게 얽혀있는 문제를 다를 때 내가 설정한 기준에 따라 판단하고 문제를 처리한다. |  |  |  |  |  |
| 20. 나는 궁금한 것이 있으면 질문하여 알려고 노력 한다. |  |  |  |  |  |
| 21. 나는 내가 잘 모르는 일들이 어떻게 이루어지는지 이해하려고 애쓴다. |  |  |  |  |  |
| 22. 나는 모르는 문제가 발견되면 알 때까지 노력한다. |  |  |  |  |  |
| 23. 나는 내가 판단하고 결정할 사항이 있으면 남에게 의존하지 않고 스스로 한다. |  |  |  |  |  |
| 24. 나는 내 의견에 대한 비판을 기꺼이 받아들인다. |  |  |  |  |  |
| 25. 나는 어떤 판단이나 결정을 내릴 때 서둘러 결론짓는 편이다. |  |  |  |  |  |
| 26. 나는 내 의견이나 다른 사람의 의견을 공평하게 평가한다. |  |  |  |  |  |
| 27. 나는 어떤 문제를 해결해 나갈 때 나 자신의 추론 능력을 믿는다. |  |  |  |  |  |

**연구 대상자 사전 사후 설문지**

- 다음은 해당질환 관련 이론적 지식을 측정하는 문항입니다. 적절한 답에 O 표 하세요.

**1. 상부위장관 출혈의 원인 중 하나인 소화성궤양을 악화시키는 요인이 아닌 것은?**

1) 벽세포수 감소

2) 미주신경 자극 증가

3) 가스트린의 과다분비

4) Helicobacter pylori 감염

5) 비스테오리드 항염증제 사용

**2. 상부위장관출혈의 원인질환중 하나인 소화성 궤양 환자에게 금연하도록 하기 위해 가장 적절한 설명은?**

1) 흡연은 식도와 폐질환을 유발한다.

2) 흡연은 위점막 자극으로 통증을 유발한다.

3) 흡연은 미주신경 자극으로 위운동을 억제한다.

4) 흡연은 식욕을 감퇴시켜 영양부족을 초래한다.

5) 흡연은 췌장의 중탄산염의 분비를 감소시켜 십이지장 내의 산도를 증가시킨다.

**3 소화성 궤양에 의한 환자에게 미주신경절제술을 하는 목적은?**

1) 유문이완

2) 위경련 조절

3) 위 운동 강화

4) 위산분비 억제

5) 식도협착 조절

**4. 소화성 궤양의 합병증에 대한 설명으로 옳지 않는 것은?**

1) 천공시 비위관 흡인으로 복강으로의 유출을 방지한다.

2) 궤양과 치유의 반복으로 유문부폐색이 발생할 수 있다.

3) 출혈이 있는 경우 토혈, 혈변, 현기증, 저혈압 등이 나타난다.

4) 출혈시 차가운 생리식염수로 비위관을 통해 위세척을 시행한다.

5) 중앙상복부에서 시작하는 예리한 통증, 판자같이 단단한 복부인 경우 천공을 의심한다.

**5. 소화성 궤양의 합병증인 출혈에 대한 치료 및 간호의 내용이 아닌 것은?**

1) 필요시 수혈을 시행한다.

2) 내시경을 통해 출혈성 병변을 소작한다.

3) Vasopressin투여하여 급성 출혈을 조절한다.

4) 금식시키고 쇼크를 예방하기 위해 정맥으로 수액을 공급한다.

5) 비위관을 삽입하여 위팽만을 감소시키고, 차가운 생리식염수로 위세척을 한다.

**6. 소화성 궤양 환자에게 복용이 허용되는 약 은?**

1) Aspirin

2) Salicylate

3) Ibuprofen

4) Corticosteroid

5) Acetaminophen

**7. 소화성 궤양을 예방하기 위한 목적으로 cimetidine(Tafamet)을 투여하는 환자에게 약물의 상호작용을 고려하지 않아도 되는 것은?**

1) Warfarin

2) Phenytoin

3) Propranolol

4) Furosemide

5) Theophylline

**8. 소화성 궤양 치료에 히스타민 2 수용체 차단제 약물을 사용하는 이유는?**

1)위산중화

2)염산분비억제

3)펩신분비억제

4)가스트린 분비억제

5)헬리코박터균 감염 치료

**9. 소화성 궤양 환자 간호로 옳은 것은?**

1) 섬유질 섭취를 늘려 위장관 건강을 도모하도록 한다.

2) 우유 섭취를 권장하여 산을 중화하도록 한다.

3) 상당히 힘든수준의 운동을 통해 위배출 속도를 증가시킨다.

4) NSAIDs 약물로 통증을 조절한다.

5) 커피, 초콜릿, 카페인 함유 음식을 피한다.

**10. 20년 전부터 위궤양을 앓고 있는 환자가 다음 증상을 호소하며 입원하였다. 가장 필요한 중재는?**

| WBC 22,000/mm3  상복부의 강한 통증  오심, 구토  호흡과 맥박증가, 혈압증가 |
| --- |

1) 경구로 수분을 섭취하게 한다.

2) 섭취량과 배설량을 사정한다.

3) 복와위를 취해준다.

4) 따뜻한 찜질팩을 복부에 적용한다.

5) 복부를 마사지 해준다

| - 임상술기 수행능력   이 검사는 평소 여러분의 위장관 출혈환자 임상판단에 대한 임상술기 수행능력을 파악하기 위한 것입니다. 각 문항은 **옳고 그른 답이 없으므로** 문제를 잘 읽고 **자신이 수행할수 있는지에 대해 생각해보고 해당부분에** O표나 √표를 하세요 | | | | |
| --- | --- | --- | --- | --- |
| **번호** | **항목** | **잘함**  **2** | **보통**  **1** | **못함**  **0** |
| 1 | 환자에게 자기소개 및 개방적 질문을 통해 환자를 확인할 수 있다. |  |  |  |
| 2 | 환자의 주호소를 파악한다. |  |  |  |
| 3 | Hematemesis/hemoptysis 를 구분할 수 있다. |  |  |  |
| 4 | PQRST 척도로 복부 통증정도를 사정할 수 있다. |  |  |  |
| 5 | 복부 장음을 청진하고 항진과 저하를 구분할 수 있다. |  |  |  |
| 6 | 오전 혈액검사 결과를 확인할 수 있다(Hgb) |  |  |  |
| 7 | 복용중인 약물이 엔세이드 계열인지 확인할 수 있다. |  |  |  |
| 8 | Hepatitis 과거력을 확인하는 이유를 설명할 수 있다. |  |  |  |
| 9 | 적절한 체위로 DRE를 수행할 수 있다. |  |  |  |
| 10 | 대상자의 불안정도를 사정하여 정서적 지지간호를 수행할 수 있다. |  |  |  |
| 11 | Nausea/Vomiting여부를 확인 할 수 있다. |  |  |  |
| 12 | 담당의에게 환자의 상황에 대해 SBAR에 의해 보고할 수 있다. |  |  |  |
| 13 | 금식의 필요성을 교육할 수 있다. |  |  |  |
| 14 | 처방에 따라 수액의 속도를 조절할 수 있다. |  |  |  |
| 15 | 오심, 구토, 토혈 등 상황에 따라 적절한 체위를 적용할 수 있다. |  |  |  |
| 16 | 수혈전 검사(ABO type, Antibody screening test, Crossmatching)에 대해 알고 채취할 수 있다. |  |  |  |
| 17 | 수혈 전 약물을 정확히 injection할 수 있다. |  |  |  |
| 18 | 수혈을 핵심기본간호술 절차에 따라 정확히 수행할 수 있다. |  |  |  |
| 19 | PPI제제를 처방에 맞게 정확히 주입할 수 있다. |  |  |  |
| 20 | 대상자에게 영양수액요법을 수행할 수 있다. |  |  |  |
| 21 | 필요시 저농도의 산소를 투여할 수 있다. |  |  |  |
| 22 | 대상자에게 소화성 궤양의 원인, 증상에 대해 설명할 수 있다. |  |  |  |
| 23 | 소화성 궤양과 출혈가능성간의 관계를 설명할 수 있다. |  |  |  |
| 24 | 소화성궤양에 사용하는 약물의 효과에 대해 설명할 수 있다. |  |  |  |
| 25 | 위장관 출혈환자의 운동과 식이에 대해 설명할 수 있다. |  |  |  |
| 26 | 간호수행후 간호평가(출혈유무, V/S, labo 등 재확인)를 수행할 수 있다. |  |  |  |
| 27 | 모든 간호과정 종료후 적절히 간호기록을 할 수 있다. |  |  |  |

| - 위장관 출혈환자 간호과정 수행에 대한 자신감   이 검사는 평소 여러분의 위장관 출혈환자 임삼판단에 대한 자신감을 파악하기 위한 것입니다. 각 문항은 **옳고 그른 답이 없으므로** 문제를 잘 읽고 해당부분에 대한 **자신의 자심감 여부를 생각해보고 해당부분에** O표나 √표를 하세요 | | | | | | |
| --- | --- | --- | --- | --- | --- | --- |
| **번호** | **항목** | **전혀 자심없다** | **자신없다** | **보통이다** | **자신있다** | **매우자신있다** |
| 1 | 위장관 출혈환자 임상판단에 필요한 객관적, 주관적 사정자료를 파악할 수 있다. |  |  |  |  |  |
| 2 | 위장관출혈환자 간호시 **가능한 임상판단**을 파악할 수 있다. |  |  |  |  |  |
| 3 | 위장관출혈환자 간호시 **가능한 임상판단 중 우선순위**를 파악할 수 있다. |  |  |  |  |  |
| 4 | 위장관 출혈환자의 임상판단에 적절한 **간호계획을 제시**할 수 있다. |  |  |  |  |  |
| 5 | 위장관 출혈환자의 간호계획에 **적절한 간호를 수행**할 수 있다. |  |  |  |  |  |
| 6 | 위장관 출혈환자의 **간호중재 수행시 우선순위**에 따라 수행할수 있다. |  |  |  |  |  |
| 7 | 위장관 출혈환자의 간호중재에 대한 **간호평가**를 할 수 있다. |  |  |  |  |  |

**수고 많으셨습니다. 감사합니다.**
