# Supplementary material for: Clinical judgment model-based nursing simulation scenario for patients with upper gastrointestinal bleeding: A mixed methods study
Source: PLoS One. 2021 May 3;16(5):e0251029. doi: 10.1371/journal.pone.0251029 (PMC8092762; doi:10.1371/journal.pone.0251029)
Supplement: S2 File — (DOCX) [file pone.0251029.s002.docx]

**Research Participant General Characteristics Pre-survey**

| -Self-determined motivation for learning-  This study is intended to examine the motivations for learning at school and home. **There is no right or wrong answer** for each question, so please read each question carefully, and **mark O or √ in the blank space that represents you the best.** | | | | | | |
| --- | --- | --- | --- | --- | --- | --- |
| No. | **Questionnaire** | Never | Seldom | Sometimes | Frequently | Always |
|  |  | 1 | 2 | 3 | 4 | 5 |
| 1 | I must study because the subjects I learn in school are important |  |  |  |  |  |
| 2 | Solving a problem is fun |  |  |  |  |  |
| 3 | There are important subjects related to the Nursing Licensure Examination |  |  |  |  |  |
| 4 | I enjoy solving difficult challenges |  |  |  |  |  |
| 5 | I think studying is a necessary part of my life |  |  |  |  |  |
| 6 | I enjoy being able to study well |  |  |  |  |  |
| 7 | I want to learn new things |  |  |  |  |  |
| 8 | I like to think |  |  |  |  |  |
| 9 | Because studying is useful for me in the future |  |  |  |  |  |
| 10 | It is fun to expand my knowledge |  |  |  |  |  |
| 11 | It’s a subject I want to understand |  |  |  |  |  |
| 12 | Overcoming failures or mistakes is rewarding |  |  |  |  |  |

| -Metacognition scale-  This study is intended to determine what learning methods you frequently use when you think, approach, and analyze the process while learning or performing tasks (metacognitive scale). **There is no right or wrong answer** for each question, so please read each question carefully and **mark O or √ in the blank space you think represents you the best**. | | | | | | |
| --- | --- | --- | --- | --- | --- | --- |
| No. | **Questionnaire** | Never | Seldom | Sometimes | Frequently | Always |
|  |  | 1 | 2 | 3 | 4 | 5 |
| 1 | Before I start studying, I think about what and how to study. |  |  |  |  |  |
| 2 | Even when I concentrate on studying, there are times when I ask myself what questions I need to learn now. |  |  |  |  |  |
| 3 | When I run out of time while reading, I only read the important parts. |  |  |  |  |  |
| 4 | I start studying after deciding the order of the subjects I will study. |  |  |  |  |  |
| 5 | While I am studying, I frequently make sure I understand the question. |  |  |  |  |  |
| 6 | I study by allotting time according to the study questions and quantity of materials. |  |  |  |  |  |
| 7 | Before I start, I decide how many materials I will study in advance. |  |  |  |  |  |
| 8 | After solving problems, I grade them and study the incorrect ones again. |  |  |  |  |  |
| 9 | When I read a book and run out of time, I skip the unimportant parts. |  |  |  |  |  |
| 10 | Before I take an exam, I plan ahead and study. |  |  |  |  |  |
| 11 | After doing my homework, I double-check to see if I did it well. |  |  |  |  |  |
| 12 | I study subjects that I am not good at or that I am not confident in |  |  |  |  |  |
| 13 | I set my study and play times and follow my schedule. |  |  |  |  |  |
| 14 | When I test myself, I review my answers solving all the problems. |  |  |  |  |  |
| 15 | I revisit questions that I do not understand well. |  |  |  |  |  |
| 16 | When I study for an exam, I only find the important parts and study those if time is short. |  |  |  |  |  |
| 17 | When studying for an exam at the end of the school year, I look for previous tests and study them. |  |  |  |  |  |
| 18 | When I am stuck on a question, I try to solve it with another approach. |  |  |  |  |  |
| 19 | I find it difficult to study efficiently without making a time plan. |  |  |  |  |  |
| 20 | When I am having trouble answering a question, I reread the textbook. |  |  |  |  |  |
| 21 | I read and think about questions that I don’t understand well. |  |  |  |  |  |
| 22 | When I study for exams, I study by making time plans for each subject. |  |  |  |  |  |
| 23 | After taking a test, I focus on studying the problems I got wrong. |  |  |  |  |  |
| 24 | When I study for exams, I pay more attention to difficult subjects. |  |  |  |  |  |
| 25 | I start studying by planning how long and how much I will study. |  |  |  |  |  |
| 26 | I think about questions again to see if I understood the topics I learned in class correctly. |  |  |  |  |  |
| 27 | When I am not sure about something while studying, I thoroughly reread the previous section’s question. |  |  |  |  |  |
| 28 | I plan each hour to study effectively. |  |  |  |  |  |
| 29 | I reflect on whether I am studying for the exam as planned. |  |  |  |  |  |
| 30 | I mark the parts I don’t understand well in class and study later. |  |  |  |  |  |

| -Critical thinking-  This study is intended to measure your tendency to think critically. **There is no right or wrong answer** for each question, so please read the question carefully and **mark O or √ in the blank space you think represents you the best**. | | | | | |
| --- | --- | --- | --- | --- | --- |
| No. | Never | Seldom | Sometimes | Frequently | Always |
| 1. When I make a decision, I tend to jump to conclusions without thinking enough. |  |  |  |  |  |
| 2. I have my own basis for my beliefs. |  |  |  |  |  |
| 3. I am willing to admit what I did wrong. |  |  |  |  |  |
| 4. I accept a situation if it turns out to be true, even if it is different from what I think. |  |  |  |  |  |
| 5. I withhold my judgment and ponder until sufficient evidence has been obtained. |  |  |  |  |  |
| 6. I think every argument needs a valid basis to support it. |  |  |  |  |  |
| 7. I often rethink what I do on a daily basis as if it were new. |  |  |  |  |  |
| 8. When I disagree with someone else’s opinion, I explain why. |  |  |  |  |  |
| 9. I am generally logical from start to end when making conclusions. |  |  |  |  |  |
| 10. Sometimes, I think about questions, even if they are from books. |  |  |  |  |  |
| 11. People say I am logical. |  |  |  |  |  |
| 12. Sometimes, I wonder if the things I believe to be right are wrong. |  |  |  |  |  |
| 13. I try to know what I don’t know. |  |  |  |  |  |
| 14. I systematically apply the problem-solving process when there is a problem to be solved. |  |  |  |  |  |
| 15. I believe I can overcome difficulties by myself. |  |  |  |  |  |
| 16. I often question what people take for granted. |  |  |  |  |  |
| 17. I am willing to try and solve complicated problems. |  |  |  |  |  |
| 18. I do not come to a conclusion right away, but I think about it repeatedly. |  |  |  |  |  |
| 19. I make decisions and solve complex problems according to the criteria I set. |  |  |  |  |  |
| 20. If I have questions, I try to ask them and find answers. |  |  |  |  |  |
| 21. I try to understand how things I do not know are done. |  |  |  |  |  |
| 22. If I encounter a problem I do not know, I try to study it until I know the answer. |  |  |  |  |  |
| 23. I do not rely on others but make decisions by myself. |  |  |  |  |  |
| 24. I am willing to accept criticisms about my opinions. |  |  |  |  |  |
| 25. I tend to jump to conclusions when making decisions or passing judgment. |  |  |  |  |  |
| 26. I value my opinions and those of others fairly. |  |  |  |  |  |
| 27. I believe in my ability to reason when I solve problems. |  |  |  |  |  |

**Research Participant**

**Pre- and Post-survey**

- The following questions measure your theoretical knowledge related to diseases. Mark the right answer.

**1. Choose a factor that does not aggravate peptic ulcers. What is one of the causes of upper gastrointestinal bleeding?**

1) Reduction of parietal cell count

2) Increased vagus nerve stimulation

3) Gastrin hypersecretion

4) Helicobacter pylori infection

5) Use of non-steroidal anti-inflammatory drugs

**2. What is the most appropriate explanation as to why patients with peptic ulcers quit smoking? Which among these is a cause of upper gastrointestinal bleeding?**

1) Smoking causes esophageal and lung disease.

2) Smoking causes pain by irritation of the gastric mucosa.

3) Smoking suppresses gastric movement by stimulation of the vagus nerve.

4) Smoking decreases appetite and causes malnutrition.

5) Smoking decreases the secretion of bicarbonate in the pancreas and increases acidity in the duodenum.

**3. What is the purpose of vagus nerve resection for patients with peptic ulcers?**

1) Pylori relaxation

2) Controlling stomach cramps

3) Strengthening stomach movements

4) Suppressing gastric acid secretion

5) Controlling esophageal stricture

**4. Which among these complications of peptic ulcer is wrong?**

1) When perforating, prevent leakage into the abdominal cavity by aspirating the nasogastric tube.

2) Pyloric obstructions may occur due to repeated ulcers.

3) If there is bleeding, hematemesis, hematochezia, dizziness, and low blood pressure.

4) When bleeding, perform gastric lavage through a nasogastric tube with a cold saline solution.

5) In case of sharp pain that starts in the upper-middle abdomen and a stiff abdomen, suspect perforations.

**5. Which among these is not a treatment method for bleeding? Which among these is a complication of peptic ulcer?**

1) Perform blood transfusions when necessary.

2) Cauterize a hemorrhagic lesion through an endoscope.

3) Control acute bleeding by administering vasopressin.

4) Have the patient fast and give them intravenous fluids to prevent shock.

5) Induce gastric distention by inserting a nasogastric tube, and wash the stomach with a cold physiological saline solution.

**6. Which of the following drugs can be taken by peptic ulcer patients?**

1) Aspirin

2) Salicylate

3) Ibuprofen

4) Corticosteroid

5) Acetaminophen

**7. Which among these is not necessary when considering drug interactions in patients receiving cimetidine (Tagamet) to prevent peptic ulcers?**

1) Warfarin

2) Phenytoin

3) Propranolol

4) Furosemide

5) Theophylline

**8. Why are histamine-2 receptor blocker drugs used to treat peptic ulcers?**

1) Neutralize gastric acid

2) Inhibit hydrochloric acid secretion

3) Inhibit pepsin secretion

4) Suppress gastrin secretion

5) Treat helicobacter infection

**9. What is the proper way to care for a peptic ulcer patient?**

1) Increase fiber intake to promote gastrointestinal health.

2) Recommend drinking milk to neutralize the acid.

3) Increase gastric emptying rate through intense exercise.

4) Control pain with NSAIDs.

5) Avoid coffee, chocolate, and other foods containing caffeine.

**10. A patient who suffered from a gastric ulcer 20 years ago complained of the following symptoms and was hospitalized. Which intervention is most needed?**

| WBC 22,000/mm^3^  Sharp pain in the upper abdomen  Nausea  Vomiting  Increased breathing and pulse  Increased blood pressure |
| --- |

1) Drink water.

2) Assess food intake and excretion amounts.

3) Lie face-down.

4) Apply a warm compress to the abdomen.

5) Massage the abdomen.

| - Clinical skill performance ability   This study is intended to determine your ability to perform clinical techniques on patients with gastrointestinal bleeding. **There is no right or wrong answer** for each question. Please read each question carefully, think about whether you can perform the technique, and **mark O or √ in the blank space that best represents your response.** | | | | |
| --- | --- | --- | --- | --- |
| **No.** | **Questionnaire** | **Good**  **2** | **Average**  **1** | **Bad**  **0** |
| 1 | I can converse with patients using self-introduction and open-ended questions. |  |  |  |
| 2 | I can obtain the patient’s home address. |  |  |  |
| 3 | I can distinguish hematemesis and hemoptysis. |  |  |  |
| 4 | I can assess the degree of abdominal pain with the PQRST scale. |  |  |  |
| 5 | I can auscultate abdominal bowel sounds and distinguish between hyperactivity and depression. |  |  |  |
| 6 | I can check the morning blood test results (Hgb) |  |  |  |
| 7 | I can check if a drug the patient is taking is an NSAID. |  |  |  |
| 8 | I can explain the need to confirm a history of hepatitis. |  |  |  |
| 9 | I can perform DRE in the proper position. |  |  |  |
| 10 | I can provide emotionally supportive nursing by assessing the subject’s instability. |  |  |  |
| 11 | I can check for nausea and vomiting. |  |  |  |
| 12 | I can use SBAR to report the patient’s condition to the attending physician. |  |  |  |
| 13 | I can educate patients on the need for fasting. |  |  |  |
| 14 | I can control the rate of IV according to the prescription. |  |  |  |
| 15 | I can move a patient to the appropriate position based on their condition, like nausea, vomiting, and hematopoiesis. |  |  |  |
| 16 | I know the pretransfusion tests (ABO type, Antibody screening test, and Crossmatching), and I can collect samples. |  |  |  |
| 17 | I can inject drugs accurately before blood transfusion. |  |  |  |
| 18 | I can perform blood transfusions accurately according to the basic nursing procedures. |  |  |  |
| 19 | I can inject PPI precisely according to the prescription. |  |  |  |
| 20 | I can perform IV nutritional therapy on the patient. |  |  |  |
| 21 | If necessary, I can administer a low concentration of oxygen. |  |  |  |
| 22 | I can explain the causes and symptoms of peptic ulcers to the patient. |  |  |  |
| 23 | I can explain the relationship between peptic ulcer and bleeding potential. |  |  |  |
| 24 | I can explain the effects of drugs used for treating peptic ulcers. |  |  |  |
| 25 | I can explain the exercise and diet needed by patients with gastrointestinal bleeding. |  |  |  |
| 26 | I can perform a nursing evaluation (reconfirmation of bleeding, V/S, and laboratory tests) after nursing, |  |  |  |
| 27 | I can make nursing records properly after completing all nursing courses. |  |  |  |

| - Confidence in performing the nursing process for patients with gastrointestinal bleeding   This study is intended to assess your confidence when making clinical judgments for patients with gastrointestinal bleeding. **There is no right or wrong answer** for each question. Please read each question carefully, think about your confidence level, and **mark O or √ in the blank space of the statement that best represents your response.** | | | | | | |
| --- | --- | --- | --- | --- | --- | --- |
| **No.** | **Questionnaire** | **Not confident at all** | **Not confident** | **Confident** | **Very Confident** | **Absolutely Confident** |
| 1 | I can objectively and subjectively identify data necessary to make clinical judgments for patients with gastrointestinal bleeding. |  |  |  |  |  |
| 2 | I can identify possible clinical judgments when caring for patients with gastrointestinal bleeding. |  |  |  |  |  |
| 3 | I can identify the priority of possible clinical judgments when nursing patients with gastrointestinal bleeding. |  |  |  |  |  |
| 4 | I can suggest an appropriate nursing plan when making clinical judgments for patients with gastrointestinal bleeding. |  |  |  |  |  |
| 5 | I can perform procedures appropriate to the nursing plan of patients with gastrointestinal bleeding. |  |  |  |  |  |
| 6 | I can perform nursing interventions for patients with gastrointestinal bleeding according to priority. |  |  |  |  |  |
| 7 | I can perform a nursing evaluation and interventions for patients with gastrointestinal bleeding. |  |  |  |  |  |

**Thank you.**
