# Supplementary material for: Clinical judgment model-based nursing simulation scenario for patients with upper gastrointestinal bleeding: A mixed methods study
Source: PLoS One. 2021 May 3;16(5):e0251029. doi: 10.1371/journal.pone.0251029 (PMC8092762; doi:10.1371/journal.pone.0251029)
Supplement: Attachment — Submitted filename: Response to Reviewers.docx [file pone.0251029.s003.docx]

March 22, 2021

César Leal-Costa, PhD

Academic Editor

*PLOS ONE*

Dear Dr. Leal-Costa,

Before anything else, we would like to thank you for allowing us to resubmit our manuscript entitled “Clinical judgment model-based nursing simulation scenario for patients with upper gastrointestinal bleeding: A mixed methods study” (Manuscript ID: PONE-D-20-40900). Moreover, we would like to thank you and the journal reviewers for your feedback, which guided us to revise and improve our manuscript.

You can find the explanations of our revisions and our responses to the reviewers’ comments immediately after this letter. The reviewers’ comments are in bold, while our responses are in normal font. Passages of text directly lifted from the manuscript are enclosed in quotation marks. We believe that the changes have improved our manuscript further and made it easier to understand.

Again, we would like to extend our sincere gratitude for your support. Please let us know if there is anything we can do to assist in the process.

We look forward to hearing from you!

Best regards,

Hyunyoung Park

College of Nursing, Chonnam University,

Gwangju, Republic of Korea

hypark@jnu.ac.kr

Reviewers’ Comments


1. Is the manuscript technically sound, and do the data support the conclusions?

The manuscript must describe a technically sound piece of scientific research with data that supports the conclusions. Experiments must have been conducted rigorously, with appropriate controls, replication, and sample sizes. The conclusions must be drawn appropriately based on the data presented.

Reviewer #1: Yes

Reviewer #2: Partly

2. Has the statistical analysis been performed appropriately and rigorously?

Reviewer #1: Yes

Reviewer #2: Yes

3. Have the authors made all data underlying the findings in their manuscript fully available?

The PLOS Data policy requires authors to make all data underlying the findings described in their manuscript fully available without restriction, with rare exception (please refer to the Data Availability Statement in the manuscript PDF file). The data should be provided as part of the manuscript or its supporting information, or deposited to a public repository. For example, in addition to summary statistics, the data points behind means, medians and variance measures should be available. If there are restrictions on publicly sharing data—e.g. participant privacy or use of data from a third party—those must be specified.

Reviewer #1: Yes

Reviewer #2: Yes

4. Is the manuscript presented in an intelligible fashion and written in standard English?

PLOS ONE does not copyedit accepted manuscripts, so the language in submitted articles must be clear, correct, and unambiguous. Any typographical or grammatical errors should be corrected at revision, so please note any specific errors here.

Reviewer #1: Yes

Reviewer #2: Yes

5. Review Comments to the Author

Please use the space provided to explain your answers to the questions above. You may also include additional comments for the author, including concerns about dual publication, research ethics, or publication ethics. (Please upload your review as an attachment if it exceeds 20,000 characters)

**Reviewer #1: The paper proposes a well-exposed and evaluated clinical simulation scenario for an urgent clinical situation. For subsequent studies it would be proposed to evaluate multiple scenarios of the same clinical situation with a randomised study design.**

Response: Thank you for your comment. Per your suggestion, the following sentences were added in the Conclusion section.

“Fifth and last, this study proposed a well-exposed and evaluated clinical simulation scenario for urgent clinical situations. Future studies can utilize a randomized study design to evaluate multiple scenarios of the same clinical situation.”

**Reviewer #2: I realize that a great work and time has been devoted to this paper. This is a topic of great significance to nursing students that can affect the learning and therefore, their future work as a nurse. So I appreciate authors examining this topic.
The paper has a lot of strengths but I think that some changes should be recommended.**

**Abstract:
Please, avoid using abbreviations in the abstract.**

Response: The abstract no longer contains any abbreviations.

**Introduction:
I suggest the authors to explain what role exactly have nursing in the approach of UGIB.
Please, write better the aim of the study.
I dont know if the aim was exactly the develop and apply of the scenario. So I think that the main aim is only the evaluation of the scenario effectiveness. So please, discuss this fact.**

Response: In response to the reviewer’s comment, the Introduction, which details the role of nursing patients with UGIB, is further described as follows.

“Understanding the clinical judgment process and how nurses think in actual clinical situations are essential considerations for simulations to be appropriately used as an alternative to clinical practice. Therefore, simulations should reinforce practicing clinical judgment by allowing students to think like a nurse. Tanner [2] presented the clinical judgment process as a model that incorporates various tasks, such as noticing, interpreting, responding, and reflecting.”

The following sentences were also added to clarify the study’s purpose.

“The study has two major purposes. First, this study aims to develop a simulation-based learning scenario integrated with the phases of Tanner’s model to create simulations that effectively improve nursing students’ capability to advance in the clinical field. Second, this study applies and evaluates a scenario’s effectiveness when caring for patients with UGIB.”

Furthermore, the effectiveness of the developed scenario is described in the first paragraph of the Discussion section.

**Methodology:
It is unclear the design of study. When the authors write “mixed methods,” do they refer to qualitative and quantitative design?**

Response: The study design’s description was revised as follows to clarify the research methodology.

“The study utilized a mixed methods design (Fig 1). Specifically, a self-report survey and video analysis were used to evaluate the developed scenario’s effectiveness and review the experimental group’s simulations, respectively [22].”

**Results:
What level, year or course were the students?
Please, explain better the sentence in line 219 “noticing 12.7 times...”**

Response:

The students’ course, year, and level were described in the Participants, Scenario development, and Verification of the effectiveness of scenario application subsections.

“Recruitment was announced on a notice board at Songwon University, and the study’s participants mainly included 3rd-year nursing students who took a course on the digestive system. The students recruited for the study understood its purpose and provided their written consent. Among the recruited students, those who had difficulties participating in discussions or team activities arising from physical or mental issues, disagreed with the confidentiality agreement related to the scenario’s operation, or took pictures and video recordings during the study were excluded.”

“The high-fidelity human patient simulation scenario was developed by two adult nursing professors and one simulation professor for 3rd-year medical school students in South Korea. This scenario mainly focused on applying clinical judgment steps (assessing, diagnosing, planning, implementing, and evaluating) in the nursing process to provide urgent care for patients with UGIB.”

“Based on the nursing domain results, the domain with the highest observed frequency compared to the expected frequency was “education” (expected = 8, observed = 5.4), and the lowest was “anxiety” (expected = 8, observed = 0.4). As observed in Tanner’s CJM phases, the phase with the highest observed frequency compared to the expected frequency was “noticing” (expected = 30. observed = 12.7), and the lowest was “reflecting” (expected = 9, observed = 2.1; Table 4).”

**Discussion:
Please, add a subheading for the Conclusions.
I suggest the authors to acknowledge at least for the students.**

Response: Per your comment, the Discussion section now has a separate Conclusions subheading. The students recruited for the study were also acknowledged in the appropriate section, which now contains the following statement.

“The study’s authors would like to express their heartfelt thanks to the gastroenterologist, nurses, professors, consultants who assisted in scenario development, the instructors and operators who assisted during scenario operation, and the participants in this study. This study could not be accomplished without their valuable time and support.”

I hope that these recommendations do not discourage the authors and I want to recommend the authors to continue working on this paper.

6. PLOS authors have the option to publish the peer review history of their article (what does this mean?). If published, this will include your full peer review and any attached files.

If you choose “no”, your identity will remain anonymous but your review may still be made public.

Do you want your identity to be public for this peer review? For information about this choice, including consent withdrawal, please see our Privacy Policy.

Reviewer #1: No

Reviewer #2: No

Additionally, the following sections were also revised based on your feedback.

**1. Journal Format: Please ensure that your manuscript meets PLOS ONE’s style requirements, including those for file naming.**

Response: The manuscript was revised to meet PLOS ONE’s style requirements, and the files attached with this manuscript are named according to the journal’s prescribed format.

**2. Methods: In your Methods section, please provide additional information about the participant recruitment method and the demographic details of your participants. Please ensure you have provided sufficient details to replicate the analyses such as:

- a description of any inclusion/exclusion criteria that were applied to participant recruitment
- a statement as to whether your sample can be considered representative of a larger population
- a description of how participants were recruited
- descriptions of where participants were recruited and where the research took place.**

Response: In response to the reviewer’s comments, the content related to the participants was revised as follows.

“Recruitment was announced on a notice board at Songwon University, and the study’s participants mainly included 3rd-year nursing students who took a course on the digestive system. The students recruited for the study understood its purpose and provided their written consent. Among the recruited students, those who had difficulties participating in discussions or team activities arising from physical or mental issues, disagreed with the confidentiality agreement related to the scenario’s operation, or took pictures and video recordings during the study were excluded.”

“To calculate the number of the study participants, G-power 3.19.2 and “ANOVA: repeated measures, within-between interaction” were chosen (effect size = 0.25, α = 0.05, power = 0.95, correlation coefficient = 0.5). The experimental and control groups included 36 students each, which was considered representative of the larger student population.”

**3. Please include additional information regarding the survey or questionnaire used in the study and ensure that you have provided sufficient details that others could replicate the analyses. For instance, if you developed a questionnaire as part of this study and it is not under a copyright more restrictive than CC-BY, please include a copy, in both the original language and English, as Supporting Information.**

Response: Two files that contain the questionnaires used for the study are included as supplementary files. The first file contains the questionnaires as initially written in Korean. The second file has the same questionnaires translated into English.

**S1 File. Original research participant presurvey questionnaire in Korean.**

**S2 File. Translated research participant presurvey questionnaire.**

.
